# Synapsin II Is Involved in the Molecular Pathway of Lithium Treatment in Bipolar Disorder

**DOI:** 10.1371/journal.pone.0032680

**Published:** 2012-02-24

**Authors:** Cristiana Cruceanu, Martin Alda, Paul Grof, Guy A. Rouleau, Gustavo Turecki

**Affiliations:** 1 McGill Group for Suicide Studies, Douglas Mental Health University Institute, McGill University, Montreal, Quebec, Canada; 2 Department of Psychiatry, Dalhousie University, Halifax, Nova Scotia, Canada; 3 Mood Disorders Centre of Ottawa, Ottawa, Ontario, Canada; 4 Centre of Excellence in Neuromics, CHUM Research Center and the Department of Medicine, University of Montreal, Montreal, Quebec, Canada; Cardiff University, United Kingdom

## Abstract

Bipolar disorder (BD) is a debilitating psychiatric condition with a prevalence of 1–2% in the general population that is characterized by severe episodic shifts in mood ranging from depressive to manic episodes. One of the most common treatments is lithium (Li), with successful response in 30–60% of patients. Synapsin II (SYN2) is a neuronal phosphoprotein that we have previously identified as a possible candidate gene for the etiology of BD and/or response to Li treatment in a genome-wide linkage study focusing on BD patients characterized for excellent response to Li prophylaxis. In the present study we investigated the role of this gene in BD, particularly as it pertains to Li treatment. We investigated the effect of lithium treatment on the expression of SYN2 in lymphoblastoid cell lines from patients characterized as excellent Li-responders, non-responders, as well as non-psychiatric controls. Finally, we sought to determine if Li has a cell-type-specific effect on gene expression in neuronal-derived cell lines. In both *in vitro* models, we found SYN2 to be modulated by the presence of Li. By focusing on Li-responsive BD we have identified a potential mechanism for Li response in some patients.

## Introduction

Bipolar disorder (BD) is a major topic in health research given its debilitating nature, lifetime prevalence and significantly high occurrence in the general population (1–2%) [1].This psychiatric condition is characterized by abnormal shifts in energy, activity levels, mood, and one's ability to carry out routine tasks. In comparison to other psychiatric conditions, BD has been shown to have relatively high heritability, with estimates ranging from 60 to 85% [1], [2]. One of the most common treatments of BD is lithium (Li), administered as metallic salts, due to its proven efficacy both as a short term intervention for manic episodes as well as a prophylactic against episode recurrence. The drug has been highly prescribed since the 1950s and 1960s when Mogens Schou showed its efficacy through a series of systematic trials with BD patients [3], and demonstrated a high success rate with approximately 30–60% of patients showing full or partial treatment response [4], [5].

Synapsin II (SYN2) is a gene that codes for a neuronal phosphoprotein involved in synaptic plasticity and transmission as well as synaptogenesis. It maps to chromosome 3p25 and has two known variants, IIa and IIb, which are highly expressed in nerve terminals in the majority of the adult brain [6] with demonstrated homology across numerous vertebrate and invertebrate organisms [7]. The majority of brain regions co-express synapsin genes at similar levels, suggesting that they are functionally complementary [8], and though all synapsins have been primarily studied for their roles in the brain, the genes' expression is widespread in the peripheral nervous system. In non-neuronal cells, synapsins are mostly found in association with the cytoskeleton, where their involvement is likely at the level of vesicular trafficking [7]. For example, Syn2 protein was isolated from rat as well as bovine chromaffin cells of the adrenal medulla [9], [10]. Though limited work has been done on SYN2 outside of neurons, expression of other synapsins has been shown in undifferentiated astrocytes [11], osteoblasts [12], liver endosomes [13], epithelial cells [14], as well as the cell lines HeLa and NIH/3T3 [15].

Given the multiple roles played by synapsins in neuronal cell function and maintenance, it may be hypothesized that disruption of these roles could result in the onset of pathological conditions. Indeed, knockout experiments have shown the absence of SYN2 to induce epileptic-like seizures in mice [16], [17] and genetic mapping identified variants in the SYN2 gene as significantly contributing to epilepsy predisposition [17], [18]. Genetic association studies have also linked SYN2 variants with schizophrenia, as shown in affected families of different genetic backgrounds [19], [20], [21]. Data for BD are more limited, however. The only reported case-control analysis of SYN2 single nucleotide polymorphisms (SNPs) in individuals with BD comes from Wang *et al.* who studied the Han Chinese population but did not find any significant association [22]. Additional work has been reported for SYN2 at the protein or mRNA levels, where several studies showed significant dysregulation in alcoholism, Huntington's disease, and schizophrenia [23], [24], [25]. In BD, Vawter *et al.* showed differential down-regulation of SYN2 protein levels in hippocampi of patients compared to non-psychiatric controls. We have recently published a linkage study in families ascertained through Li-responsive BD probands, where the SYN2 gene was identified as one of the more interesting candidates [26]. In the same study, at the mRNA expression level, SYN2 was shown to be up-regulated in the prefrontal cortex of patients [26]. In the present study, we hypothesize that the implication of SYN2 in BD is more prominent in a subset of BD patients. Moreover, we predict that in such patients SYN2 is more relevant to the response to lithium treatment.

To explore these hypotheses, we conducted a series of studies investigating the expression of SYN2 in BD, particularly as it pertains to lithium treatment. Because this candidate gene was originally identified through a linkage study of lithium-responsive BD families, we investigated what effect lithium treatment would have on the expression of SYN2. We performed *in vitro* long-term treatment studies in Epstein-Barr-virus transformed lymphoblastoid cell lines (LCLs) from BD patients characterized for excellent Li-response (as described previously) [27], [28], [29] in order to identify the effect of this drug in a model replicating the genetic background of response. In addition, we performed the same experiments with human neuroblastoma and glioblastoma cells to model the biological context.

## Materials and Methods

### I. Ethics statement

Ethics approval for the use of human samples in this study was obtained from the Capital District Health Authority (CDHA) in Halifax, Nova Scotia. All subjects gave written informed consent to their participation in the study in regards to sample collection and the generation of lymphoblastoid cell lines; no subjects had reduced capacity to consent. Sample collection and cell lines generation has been described previously [29], [30].

### II. BD Li-response lymphoblastoid samples

Subjects were diagnosed with BD I and BD II according to both Research Diagnostic Criteria (RDC) and DSM-IV criteria, and followed prospectively at specialized clinics in Hamilton, Ottawa and Halifax [27]. Their clinical course was characterized by a high number of manic and depressive episodes before Li treatment. The responders (n = 11) showed full stability on long-term Li monotherapy. The non-responders (n = 12) continued experiencing illness episodes in spite of good compliance documented by therapeutic blood levels. These are the same criteria as outlined previously [27], [28]. Unaffected controls (n = 13) were matched for ethnic background and excluded if they had a history of BD, schizophrenia, or major depression. Peripheral blood samples were obtained from patients and controls following standard procedures and Epstein-Barr virus-transformed β-lymphoblastoid cell lines were generated as described previously [29], [30].

### III. Cell culture

To determine patient-specific effects of Li on target genes, *in vitro* assays were performed in LCLs from excellent Li-responders, non-responders, and healthy controls. Aliquots of frozen cell lines were stored in liquid nitrogen after Epstein-Barr virus transformation for each sample according to “LCL frozen storage” time until all samples were randomized, thawed for experiments, grown and processed in a sequential fashion as described below. This effectively ensures no difference in passage number between LCL samples and no batch effect. Cells were cultured in Iscove's Modified Dulbecco's Medium (IMEM) supplemented with 15% FBS, 1% Fungizone and 1% penicillin/streptomycin/glutamine (Invitrogen) in a 5% CO_2_ humidified incubator at 37°C, in the continuous presence of 1.0 mM LiCl or vehicle (NaCl) for 7 days [29] after which cell pellets were collected and frozen at −80°C. Experiments were performed in triplicate. Clinical and demographic characteristics of patient and control LCLs are listed in Table 1.

**Table 1:** Lymphoblastoid cell line sample group demographics.

	Controls (C)	Responders (R)	Non-Responders (N)	Group differences (p≤0.05)
Subjects (M/F)	13 (3/10)	11 (5/6)	12 (3/9)	Not Significant
Age at DNA sampling (yr)	31±4.7	53.5±4.3	47.9±3.8	C vs. R and C vs. N
LCL frozen storage (yr)	3.7±0.3	7.8±1.0	6.8±0.5	C vs. R and C vs. N
Age at onset (yr)	n/a	32.6±3.5	29.8±3.5	Not Significant

Data are presented as mean±SEM for non-psychiatric controls, bipolar disorder patients who are excellent lithium responders (“Responders”) and bipolar disorder patients who do not respond to lithium treatment (“Non-Responders”). “Age at sampling” refers to the subject's age at the time blood was drawn. “LCL frozen storage” refers to the length of time of liquid nitrogen storage after Epstein-Barr-Virus transformation. “Age at onset” refers to the age at which patients were diagnosed with BD.

To determine cell-type-specific modulation of candidate genes in the brain, *in vitro* assays were performed in three cell lines: HEK293 (human embryonic kidney, ATCC CRL1573) as a non-brain control, SK-N-AS (human neuroblastoma, ATCC CRL2137), and U-118 MG (human glioblastoma; astrocytoma, ATCC HTB15). Cells were cultured at 37°C in Dulbecco's Modified Eagle Medium (DMEM) supplemented with 10% FBS, 100 U/ml penicillin and 100 µg/ml streptomycin (Invitrogen) in a 5% CO_2_ humidified incubator at 37°C. For Li treatments, cells were grown in the continuous presence of 0.5 mM, 1.0 mM, or 2.0 mM LiCl or vehicle (NaCl) for 7 days after which cell pellets were collected and frozen at −80°C. Experiments were performed in triplicate.

### IV. Real-time PCR

Total RNA was extracted from frozen cell pellets using the RNeasy Mini Kit (QIAGEN). For synthesis of cDNA, M-MLV reverse transcriptase (Gibco, Burlington, Ontario) and oligo(dT)16 primers (Invitrogen) were used. Real-time PCR reactions were run in quadruplicate using an ABI PRISM 7900HT Sequence Detection System (Applied Biosystems) and the Power SYBR® Green PCR Master Mix (Applied Biosystems). Relative expression was calculated using the relative quantitation method (ΔΔCt) in the RQ Manager 1.2 software (Applied Biosystems) with GAPDH as an endogenous control.

### V. Data analysis

Test coefficients and probability distributions were calculated using statistical software GraphPad Prism 5 and SPSS.

## Results

### Lithium affects gene expression in transformed lymphoblastoid cell lines (LCLs) distinctly in lithium responders compared to both non-responder BD patients and controls

**Figure 1:**
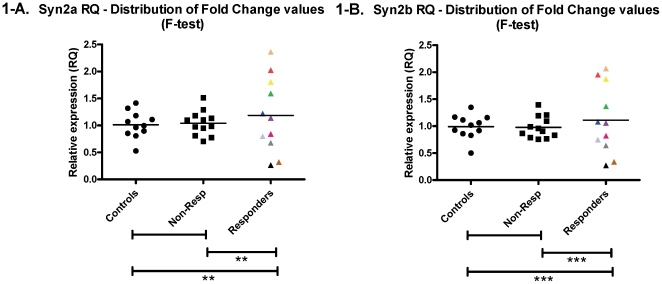
Lymphoblastoid cell line expression. Relative Quantification (RQ) values from qRT-PCR relative to GAPDH as an endogenous control. The groups compared are non-psychiatric controls, bipolar disorder patients without positive response to lithium (Non-Resp) and bipolar disorder patients with excellent response to lithium. The expression analyses were performed with separate primer sets for SYN2a (left) and SYN2b (right). The asterisks refer to F-test p-values depicting the differences in distribution between the individual expression changes in each group (** p-value≤0.001; *** value≤0.0001). There were no significant mean group differences, as indicated in Table 2.

To determine patient-specific effects of Li on the target genes, *in vitro* assays were performed in Human Epstein-Barr virus–transformed LCLs from excellent Li-responders (R), non-responders (N) and controls without psychiatric history (C) [30]. For long-term treatment, cells were cultured in the continuous presence of 1.0 mM treatment (LiCl) or vehicle (NaCl) for 7 days [29]. Data in Figure 1 are presented as fold change between Li treatment and vehicle treatment values. We performed a ANCOVA analyses with “Age at Sampling” and “LCL frozen storage” as covariates, followed by Tukey's multiple comparison post-tests for group comparisons, but found no significant mean differences between the three groups: C vs. R, C vs. N, and R vs. N for either Synapsin II variant (SYN2a p = 0.613, SYN2b p = 0.691), as shown in Table 2.

**Table 2:** Lithium response in lymphoblastoid cell line samples.

	*SYN2a*			*SYN2b*		
	Control/Non-Responder	Control/Responder	Non-Responder/Responder	Control/Non-Responder	Control/Responder	Non-Responder/Responder
ANCOVA p-value	0.867			0.916		
Tukey's Test	0.231	1.315	1.112	0.108	0.993	1.123
F-test	0.897	0.001**	0.001**	0.839	0.0009***	0.0008***

ANCOVA analysis was performed to compare the three groups (Controls, Responders, and Non-responders to lithium treatment) separately for Syn2a and Syn2b expression. The variables “Age at sampling” and “LCL frozen storage” were used as covariates.

Interestingly, there was a significant difference in the distribution of expression fold-change in the responder patient group as compared to the non-responders and the controls. LCLs from non-responder BD patients displayed the same distribution pattern as the controls whereas the Li-responder patient LCLs had a broader spectrum of expression than the other two groups. The same pattern was observed with the SYN2a variant shown in Figure 1.A (F-test P = 0.001 for both C vs. R and N vs. R) as with the SYN2b variant shown in Figure 1.B (F-test P<0.001 for both C vs. R and N vs. R). Furthermore, the expression pattern was consistent across the two variants, with subjects showing consistently low or high expression in both the SYN2a and SYN2b variant. This was illustrated through the color-coding in Figure 1.

### Environmental factors do not explain the variant effect of lithium in Responders

Given the fact that in some patient LCLs both SYN2a and SYN2b were up-regulated by lithium treatment while in others the two variants were down-regulated, we attempted to elucidate the stratifying factors responsible for this behavior. Ethnic background did not differ across subjects as all were Caucasian of European descent, so this variable was not included in the analyses. We investigated a number of other factors including age of onset, initial Li prescription, and time on Li prior to DNA collection (Table 3). Furthermore, we investigated factors relating to psychiatric medication such as Li dosage and use of other medications, as well as family history of other psychiatric disorders. We determined normality of each dataset using a Shapiro-Wilk normality test and computed Pearson's correlations for normally distributed and Spearman's correlations for non-normally distributed datasets. None of the 15 potential environmental covariates showed significant correlations with either SYN2a or SYN2b expression values, demonstrating that the reported variance difference cannot be explained by these possible covariates (Table 3).

**Table 3:** Correlations of covariates with RQ expression values in excellent lithium responders.

	*Shapir- Wilk Normality*		*SYN2a RQ*			*SYN2b RQ*		
	p-value	Normal distribution	Pearson coefficient	Spearman coefficient	p-value	Pearson coefficient	Spearman coefficient	p-value
LCL frozen storage (yr)	0.2207	Yes	0.211		0.533	0.237		0.482
Age at DNA sampling (yr)	0.8805	Yes	−0.070		0.838	−0.065		0.849
Age at Onset (yr)	0.6388	Yes	0.217		0.521	0.158		0.644
Age at first treatment Li (yr)	0.3542	Yes	−0.404		0.320	−0.449		0.264
Time between onset and DNA collection	0.2446	Yes	−0.291		0.385	−0.227		0.502
Li Treatment response Score	0.2172	Yes	−0.129		0.705	−0.185		0.585
Episodes before Li	0.0114	No		−0.527	0.145		−0.527	0.145
Time on Li treatment (yr)	0.0456	No		0.477	0.194		0.477	0.194
Li dose at DNA sampling	0.5553	Yes	−0.437		0.239	−0.432		0.246
Number of other psych drugs	0.169	Yes	0.277		0.470	0.345		0.364
Family History Depression	0.0012	No		0.015	0.965		0.015	0.965
Family History Bipolar Disorder	0.0085	No		−0.193	0.569		−0.193	0.569
Family History Schizophrenia								
Family History Anxiety	<0.0001	No		0.100	0.770		0.100	0.770
Family History Alcoholism	0.0004	No		0.438	0.178		0.438	0.178

To try and explain the distribution abnormal of Syn2 expression in Li-responders we computed correlations between RQ values and 15 potential covariates relating to age at sampling, onset, treatment start, etc., lithium treatment, as well as family history of other psychiatric disorders. (No samples had any family history of schizophrenia.) Normality of distribution was determined using the Shapiro-Wilk normality test and correlations were determined using Pearson's or Spearman's tests accordingly. No significant correlations were found with any of these variables.

### Synapsin II shows cell-type specific response to lithium treatment in neuroblastoma cells

**Figure 2:**
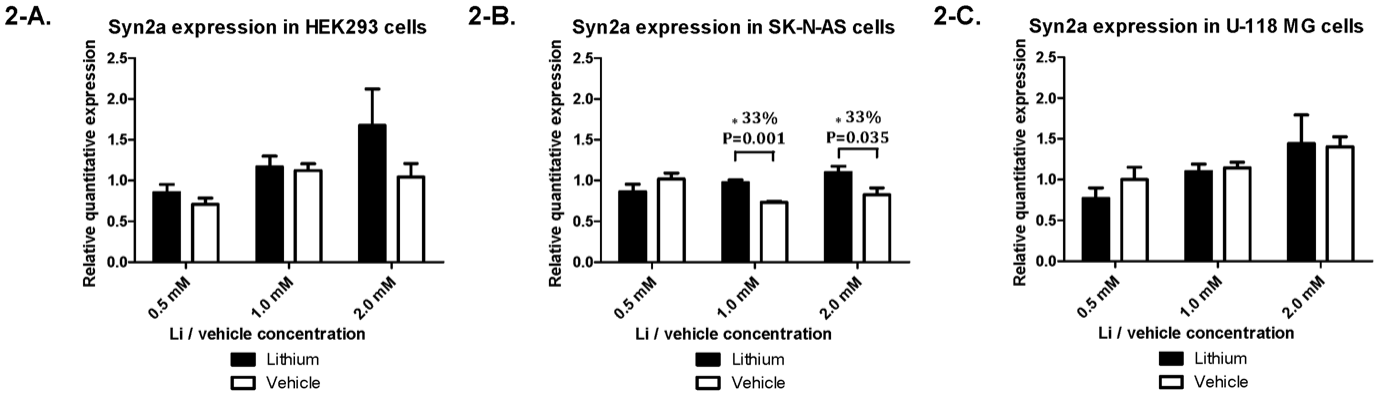
Cell lines expression for SYN2a. Expression in (A) HEK293 embryonic kidney cells, (B) SK-N-AS neuroblastoma cells, and (C) and U-118 MG glioblastoma/astrocytoma cells for the Synapsin IIa variant compared to GAPDH. P-values depicting the mean differences between 3 independent experiments for each cell line at each of the 3 treatment concentration of either lithium or vehicle (0.5 mM, 1.0 mM, and 2.0 mM).

**Figure 3:**
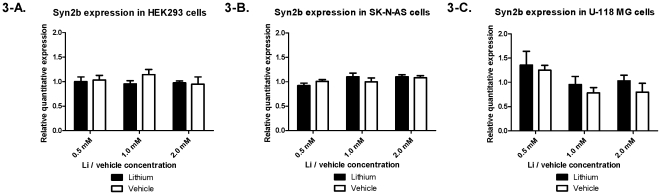
Cell lines expression for SYN2b. Expression in (A) HEK293 embryonic kidney cells, (B) SK-N-AS neuroblastoma cells, and (C) and U-118 MG glioblastoma/astrocytoma cells for the Synapsin IIb variant compared to GAPDH. P-values depicting the mean differences between 3 independent experiments for each cell line at each of the 3 treatment concentration of either lithium or vehicle (0.5 mM, 1.0 mM, and 2.0 mM).

Since our previously reported brain expression results [26] were from homogenate tissue brain extracts, we set out to investigate a possible cell-type-specific effect of lithium treatment. As such, we used three cell lines representing neurons (SK-N-AS), glial cells (U-118 MG) and embryonic kidney cells as a non-central nervous system cell control (HEK293). In order to detect concentration-specific effects, three different concentrations of treatment (LiCl) or vehicle (NaCl) were used: 0.5 mM, 1.0 mM, and 2.0 mM – the values represent lower and higher ends of the therapeutic concentrations of lithium used clinically. SYN2a demonstrated a significant 33% increase in expression when treated with LiCl compared to vehicle at both of the two higher treatment concentrations: 1.0 mM and 2.0 mM (P = 0.001 and 0.035, respectively) in the neuronal cell line (Figure 2). A similar data set was collected for the SYN2b variant, but this had no significant change in expression in any of the conditions tested (Figure 3), suggesting that our findings are specific to SYN2a.

## Discussion

Synapsin II is a candidate gene that was originally identified through a linkage study of Li-responsive BD families. This gene was also shown to be dysregulated in the post-mortem brains of patients with BD as compared to psychiatrically healthy controls in the same study [26]. Thus, we were interested to investigate the effect of Li treatment on the expression of this gene. We did so in the genetic context of the disorder by treating with Li monotherapy Epstein-Barr virus-transformed lymphoblastoid cell lines from BD patients characterized as excellent Li-responders or non-responders, as well as healthy controls with no history of psychiatric disorders. We found that the pattern of expression was significantly different in Li-responders compared to both non-responder BD patients as well as controls. However, the direction of change of expression was not uniform across subjects (Figure 1), resulting in no overall mean differences between groups. These data suggest that Li modulates SYN2 expression in a way that is specific to Li-responders, possibly reflecting significant genetic heterogeneity.

The relevance of SYN2 expression in peripheral cells compared to the central nervous system in BD patients is not clear from our findings, particularly since we saw no mean differences between Li-responders, non-responders, and controls. It is however clear from the literature that the gene is expressed, though at more basal levels, in lymphoblasts as well as many other cell types. Despite their peripheral origin, studying transformed LCLs offers the benefit of performing *in vitro* assays on cells from patients and studying putative factors in their endogenous expression context. However, results from these experiments should be considered with a level of scepticism, as the relevance of SYN2 expression in this cell type is unclear.

Environmental factors could be involved in Li's regulatory role, which might account for the observed patient-specific effects in Li-responders. To investigate this possibility we computed correlations with a number of environmental factors relating to age of patients, Li therapy, and family history of other psychiatric disorders (for a complete list, refer to Table 3). However, none of the potential covariates correlated with SYN2a or SYN2b expression values, suggesting that the source of variation may be related to genetic or possibly epigenetic differences between patients. For example, variants in CREB genes [31] or GSK3B [32], have been shown to associate with Li-treatment response. Similarly, it is possible that epigenetic factors may increase SYN2 expression variance among patients. Though this is of interest, to our knowledge, no studies have investigated the role of Li treatment on epigenetic modifications in the human brain. However, valproate, another widely used mood stabilizer, is well known for its inhibitory effect on histone deacetylases (HDACs) [33], [34] and therefore, it is possible that at least part of Li's action may be related to epigenetic regulation. Another epigenetic regulatory level where lithium's effect could be confounded is microRNA-mediated regulation. Studies in LCLs [35] and animal models [36] have shown the drug's global effect on this class of molecules. For a variety of biological reasons, each patient's LCLs could be enriched in a combination of regulatory factors which could then impact the response to Li treatment.

Since our LCL results do not automatically represent what is occurring in the brain, we sought to determine if Li would have a cell-type-specific effect on SYN2 expression in model cell lines representative of the brain, and showed a significant change in the neuronal cell line SK-N-AS only (Figures 2 and 3). There was an effect at 1.0 and 2.0 mM Li, but not at 0.5 mM, suggesting that this concentration was not high enough to elicit a response. Interestingly, the effect was specific to the SYN2a variant (Figure 2), as the SYN2b variant remained unchanged between conditions (Figure 3). Originally, SYN2 had been believed to display neuron-specific expression in the brain; however, further studies demonstrated the gene's expression in other cell types, though at considerably lower concentrations [37], [38]. SYN2 is expressed at basal levels in various cell types and thus lithium likely modulates its expression to a certain degree in these cells but perhaps not in a functionally-relevant manner. This is consistent with the fact that synapsins are evolutionarily conserved from humans to very primitive organisms and likely their expression has become more specialized in higher organisms through a loss of the ability to regulate other cellular functions but not necessarily through a complete loss of expression [7].

According to our results, in neurons, Li treatment significantly increases SYN2 expression perhaps by also recruiting other neuron-specific transcription factors that bind to the gene's promoter such as EGR1 (early growth response 1), which has been suggested to regulate the gene [39], or AP-2alpha, which has been shown to be regulated by lithium [40]. Our results from LCLs are seemingly contradictory, as Li has an up-regulating effect on SYN2 in some patients, and a down-regulating effect in others. To interpret these results, one needs to consider that lithium acts as a mood stabilizer in patients who present both manic and depressive episodes. These clinical episodes are characterized by symptoms that are on opposite sides of the mood spectrum. Accordingly, manic patients present mood and neurovegetative activation, while depressed patients are characterized by a decreased mood levels and neurovegetative inhibition. Therefore, in order to be an effective mood stabilizer, Li needs to act by normalizing variance.

One interesting addition to this study would have been direct evidence for the effect of Li on SYN2 expression in the central nervous system of BD patients. An ideal study would investigate the expression of SYN2 variants in the post-mortem brains of BD patients who had been excellent responders to prophylactic Li for an extended period of time, so as to match the criteria used for our LCL samples. However, post-mortem brain donors with a history of BD are most often suicide completers. The literature provides extensive evidence for the anti-suicidal effects of Li prophylaxis through observational studies [41], [42], randomized controlled studies [43], [44] and meta-analyses [45], [46]. Thus, such a study would be logistically quite challenging.

Another limitation of our study is the lack of protein-level evidence to support our mRNA-level findings. Such validation would be interesting in the pursuit of qualifying SYN2 as a factor of potential pharmacological significance. However, the results presented here mainly point to SYN2 as a new mediator of Li action. Perhaps by further investigating how SYN2 is regulated we will also elucidate lithium's mode of action. There are likely several regulatory levels at play and clarifying them will be instrumental for our understanding of lithium response in BD, but as it stands the pharmacological application of this work is preliminary.

In conclusion, this is, to our knowledge, the first study attempting to determine the effect of Li treatment on mRNA-level expression of SYN2. We found a responder-specific effect of Li in LCLs from BD patients, suggesting that even though the gene is important for BD in general, there are genetic or epigenetic differences in Li responders that make them more susceptible to modulation of SYN2. Additionally, we showed that the effect of long-term treatment with Li is likely cell-type specific. As far as brain expression, our data suggest that the effect of lithium treatment is only significant in neuronal cells and not in astrocytic or glial cells. Support from additional cell types would be important to strengthen the validity of these conclusions. Our distinct findings for the two SYN2 variants as well as the reported homology in sequence and function of the family of synapsin genes opens up the question of whether the other synapsins have a neuron-specific effect, as well as a patient-specific effect. Our study points to a very interesting player in response to Li prophylaxis, but more studies are required to decipher the full pathway of Li action that leads to its stabilizing effect in a large fraction of BD patients.
